# Validation of the Thai version of the World Health Organization’s Quality of Life Scale (WHOQOL-BREF-THAI) among Thai nursing students in northeast Thailand: A multi-centre study

**DOI:** 10.12688/f1000research.161251.1

**Published:** 2025-02-27

**Authors:** Ueamporn Summart, Monthida Sangruangake, Jumrusluk Charoensaen, Wiraporn Suebsoontorn, Metha Songthamwat

**Affiliations:** 1 Faculty of Nursing, Roi Et Rajabhat University, Kho Kaew Subdistrict, Selaphum District, Roi Et, 45120, Thailand; 2 Faculty of Nursing, Udon Thani Rajabhat University, Udon Thani, Udon Thani, Thailand; 3 Faculty of Nursing, Roi Et Rajabhat University, Ko Kaeo Subdistrict, Selaphum District, Roi Et, Thailand; 4 Faculty of Nursing, Nakhon Ratchasima Rajabhat University, Nakhon Ratchasima, Nakhon Ratchasima, Thailand; 5 Department of Obstetric and Gynaecology, Udon Thani Hospital, Udon Thani, Udon Thani, Thailand

**Keywords:** Quality of life, Nursing, Southeast Asian people, WHOQOL-BREF, Confirmatory factor analysis.

## Abstract

**Background:**

The Thai version of the World Health Organization Quality of Life Scale (WHOQOL-BREF-THAI) has been verified among a variety of populations. However, there is a lack of existing research on its comprehensive psychometric properties, specifically when used with nursing students. This study examined the WHOQOL-BREF’s unique psychometric features with Thai nursing students in northeast Thailand.

**Methods:**

In this cross-sectional study, Thai nursing students were recruited using multi-stage simple random sampling from universities located in the northeast of Thailand. The data was collected via self-assessment questionnaires, and the 3,570 participants were then randomly split into two subsamples (group 1, n=2,000, and group 2, n=1,570). After reducing the number of items using statistical methods, an exploratory factor analysis (EFA) was carried out on group 1 to explore the factor structure of the WHOQOL-BREF-THAI. Finally, group 2 was used in a confirmatory factor analysis (CFA) to validate the EFA’s modified structure along with an assessment of the construct validity of the WHOQOL-BREF-THAI.

**Results:**

Principal component analysis on a random subsample supported a four-factor model with 24 items, originally suggested for factorial construct validity; these 24 items were distributed across the original four domain. The domain structure for the purpose showed a good fit in the CFA on the other subsample. Internal reliability was satisfactory (Cronbach’s alpha was greater than 0.70) for both the total scale and subscales. In terms of convergent validity, average variance extracted (AVE) revealed that all WHOQOL-BREF-THAI subscales achieved convergence, with AVE values ranging from 0.50 to 0.69.

**Conclusion:**

These results reveal that the WHOQOL-BREF-THAI is a valid and reliable tool which health care providers can utilize to measure QOL among Thai nursing students. Therefore, the WHOQOL-BREF can serve as an essential tool for researching the factors influencing nursing students’ QOL, with implications for nursing education.

## Introduction

The concept of quality of life (QOL) ^1^ refers to a person’s perception of their current condition, considering existing cultural norms and values. This judgment includes a person’s objectives, expectations, standards, and concerns _._^2^ QOL can be defined as a comprehensive, complicated notion that includes autonomy, individual and subjective perceptions, and the integration of biopsychosocial components. ^3^ University life is one of the most difficult yet exciting phases of any person’s life. Every student must deal with a wide variety of complex situations that prepare them for their future career and life. ^4^ Many factors impact university students’ QOL, such as psychological issues, social relationships, and self-evaluation. These factors may have a major effect on the daily lives of students, their academic performance, and their overall behaviour. ^5^

Nursing students are a group of people whose QOL is being challenged. While undergoing their training, they are exposed to many stressful and difficult situations, such as assisting mothers in labour, coping with traumatic events, overcoming problems and restrictions, and ensuring that patients are able to die with dignity. ^6^ Moreover, prior studies have reported that higher stress perceptions, arising from patient care, the clinical setting, and interactions with faculty, peers, and staff, all predicted a negative QOL. ^7^ In terms of these highly stressful situations, a poor QOL may have an impact on nursing students’ learning processes or even cause them to drop out, resulting in a lower quality and standard of graduate nurses. ^8^ As a result, maintaining good QOL among nursing students is essential so that nursing instructors can help shape students into effective registered nurses.

QOL evaluation is critical for determining whether nursing students have excellent well-being levels as they progress through the learning process. Therefore, to assess the effectiveness of an intervention in increasing a person’s QOL, it is critical to use a validated test that measures all elements of life. While most QOL measures commonly evaluate how physical and mental health issues affect daily living, ^9^ the World Health Organization Quality of Life Instrument-Abbreviated Version (WHOQOL-BREF), one of the most widely used questionnaires, goes beyond evaluating medical outcomes to incorporate social and environmental aspects. ^1^ In a cross-sectional study of preclinical medical students in Saudi Arabia using the WHOQOL-BREF, strong academic achievement was positively associated with QOL, particularly in the physical health, psychological health, social relationships, and environment domains. ^10^ Similarly, it has been found that the academic success motivation and QOL domains of nursing students have relationships, especially in the psychological health domain. ^11^

The WHOQO-BREF, a shortened version of the WHOQOL-100, was developed for simplified administration and was evaluated in 23 countries, including Thailand. It comprises 24 items designed to examine QOL perceptions in four domains: physical health, psychological health, social relationships, and environment, as well as two items on overall QOL and general health. ^12^ Globally, many studies employing various target demographic samples (both general population and those with a variety of diseases) have indicated that that the WHOQOL-BREF questionnaire has excellent psychometric properties. ^13– 25^ This scale has been confirmed as a four-factor structure according to many validation studies. ^13, 23, 26^ However, inconsistency in the factorial structure via confirmatory factor analysis (CFA) still exists across different settings. Prior validation research, however, demonstrated that a two-factor model was the optimum model for the modified WHOQOL-BREF test in a large sample of teenagers in Nigeria. ^27^ Another study in Vietnam used alternative models with only three factors in young adults. ^28^ Moreover, previous research using the WHOQOL-BREF among samples of Portuguese-speaking people from three different countries on three different continents discovered distinct WHOQOL-BREF configural structures among these samples. These results suggest that the cultural context can influence the WHOQOL-BREF QOL assessment. ^17^ In addition, another study in Thailand found that an extended five-factor scale, in the medical care domain, could be utilized by health care practitioners to accurately assess the demands of women who had experienced intimate partner violence. ^29^

To date, the psychometric qualities of the Thai version of the WHOQOL-BREF have been studied in a broad range of populations, such as patients with HIV, and college students in Thailand. ^13, 29, 30^ However, comprehensive documentation of the psychometric properties, including factor structure (particularly among nursing students), in this setting is completely lacking in the literature, except for use of this tool for QOL assessment. One recent study revealed that the overall QOL of 273 nursing students was high, and the reliability of this instrument was 0.95. ^8^ Since a comprehensive review of the instrument’s psychometric properties remains lacking, these findings may not be valid for nursing students. In addition, previous evidence confirmed that, before this tool can be used with these students, its psychometric properties must first be validated for this population, especially since prior research found significant issues when the questionnaire was utilized with younger adult samples. ^6, 31, 32^

In nursing education, it is critical to measure QOL to optimize training, as stress and burnout are significant factors influencing drop-out rates. ^7, 32^ Nursing students’ QOL is also likely to have an impact on their future clinical competence. Consequently, resolving this issue requires the application of a tool designed specifically for assessing their QOL. Given these concerns, the goal of this research was to assess the psychometric properties of the WHOQOL-BREF-THAI when applied to Thai nursing students.

## Methods

### Research design

This cross-sectional study was conducted using the dataset derived from a larger multi-centre study a project entitled “The prevalence and risk factors of non-communicable diseases (NCDs) among undergraduate nursing students in Northeast Thailand”. The recruitment of student participants was carried out by research partners from participating universities, beginning on various dates between June 2024 to September 2024.

### Sample and sampling technique

The target population of this study comprised Thai nursing students nationwide, and sampling was undertaken using a multistage sampling technique. In the first stage, 15 nursing institutions were chosen using a simple random sampling technique from **23** nursing institutions in northeastern regions. In the second stage, 6,500 students were selected from the **1**5 chosen institutions using stratified proportional sampling with simple random sampling. Code numbers were created to protect the privacy of students. From a total of 6,500 students, 3,570 first- to fourth-year nursing students were sampled using simple random sampling. The inclusion criteria for participation in this study were being aged 18 years or older and a nursing student at the institute for at least 6 months during the study period. Inappropriate responses to items, such as responding to only part of the general information in the WHOQOL-BREF, served as the exclusion criteria.

***Measurement***

Respondents were obtained using self-assessment questionnaires for data collection. They were contacted, given full details about the study, and asked to participate voluntarily. There were two sections to the questionnaire. The first section comprised questions designed to obtain information about respondents’ socioeconomic characteristics, including age, gender, academic year, and lifestyle/health information (for example, smoking habits, alcohol consumption, sports and recreation activity, number of hours using electronic devices and personal medical history).

The WHOQOL-BREF-THAI was used to assess the respondents’ QOL. Permission to use the Thai WHOQOL-BREF for data collection was obtained from Bureau of Mental Health Academic Affairs: BMHA Department of Mental Health: DMH, Ministry of Public Health, Thailand. This instrument consists of 26 items, with the first two assessed and scored individually to assess the overall sense of QOL (Q1) and satisfaction with general well-being (Q2). Items 3-26 are used to assess four domains: the physical health (7 items), psychological health (6 items), social relationships (3 items), and environment domains (8 items). The two additional questions assessing overall QOL and general health were not considered to be part of any of the four domains and were thus eliminated from the factor analysis in this study. A 5-point Likert response scale is used in the instrument, so these items were scored from 1 (very poor/dissatisfied) to 5 (very good/very satisfied) on a 5-point scale. According to the guidelines, raw WHOQOL domain scores were converted to a 4-20 scale. After reversing the raw scores of Q _3_, Q _4_ and Q _26_, the domain scores were calculated by adding the scores of each domain’s items together. The scores were then linearly transformed to a 0-100 scale. Higher scores indicate a higher level of QOL. Several previous studies have demonstrated the WHOQOL-BREF’s validity and reliability. ^1^ The Cronbach’s alpha values of the physical health, psychological health, social relationships, and environment domains were 0.68, 0.71, 0.76, and 0.88, respectvely. ^1^

### Data collection

Before beginning the survey, the dean of each nursing college/university granted permission to collect data, and all participants provided informed consent. The Roi-Et Rajabhat University Ethics Committee for Human Research approved the study protocol (Reference No. RERU-EC 013/2567). The date of approval was the 15th of January 2024. All participants were informed of the study’s objectives and the confidentiality of the data, and they were assured that the data would be used solely for research purposes and that refusing to participate would have no bearing on their current or future studies. Students were not compensated or otherwise encouraged to participate in the study. Respondents were free to leave the study at any time.

### Sample size

The sample size was determined using the formula “sample size = number of items X number of participants,” which is commonly used in survey development research. Each questionnaire item should have a sample size of 5 to 10 participants. ^33^ Accordingly, our study included 3,570 nursing students from 15 universities primarily located in the northeast of Thailand. Consequently, larger sample sizes may yield more significant factor loadings and more generalizable results. The study involved nursing students aged 18 and above who were enrolled as nursing students at the institute during the study period, and incomplete items of the Thai version of the WHOQOL-BREF served as the exclusion criteria. To avoid overfitting of the models, the exploratory factor analysis (EFA) and confirmatory factor analysis (CFA) were conducted on a random split of the participants into two subsamples (group 1, n = 2,000, and group 2, n = 1,570).

### Statistical analysis

Regarding the demographic characteristics of the participants, descriptive statistics, with means and standard deviations for continuous variables and frequency and percentages for categorical data, were used.

The floor and ceiling effects of the questions were analysed by considering the percentages of respondents selecting the lowest and highest response options, respectively. In this context, items with percentages of 15% or lower were deemed free from these effects. The discriminative capacity of the items was evaluated using the corrected item-rest polyserial correlation, with acceptable indices defined as those exceeding 0.20. ^34^

The scree plot and parallel analysis (based on principal component analysis) was used with subsample group 1 to examine the number of components in the EFA for the WHOQOL-BREF measurement model. The structure of factors was further examined using primary axis factoring in conjunction with varimax rotation. Factor loadings less than 0.40 were suppressed, while item cross-loadings greater than 0.20 were deleted sequentially. Communality represents the percentage of variance in the total items accounted for by all factors. Furthermore, factor loadings were employed to compute the average variance extracted (AVE) and composite reliability (CR).

CFA was performed to evaluate the WHOQOL-BREF factorial models. The weighted least squares means, and variance adjusted (WLSMV) approach was used to perform CFA on the polychoric correlation matrix. ^35^ The model’s fit was evaluated using goodness-of-fit indices (χ ^2^/df ), Tucker-Lewis index (TLI), comparative fit index (CFI), and root mean square error of approximation (RMSEA). Values of χ ^2^/df ≤ 5.00, CFI and TLI ≥ 0.90, and RMSEA ≤ 0.10 indicate an acceptable fit, while values of χ ^2^/df ≤ 2.00, CFI and TLI ≥ 0.95, and RMSEA ≤ 0.05 indicate a good fit. ^36^

Reliability analysis was carried out using internal consistency reliability for each dimension. Internal consistency was assessed using Cronbach’s alpha coefficient. Internal consistency reliability was evaluated using CR and Cronbach’s alpha. When the values for the four subscales are greater than 0.60, CR is considered acceptable, and a Cronbach’s alpha value greater than 0.70 for all subscales is considered satisfactory. ^35^ Likewise, the Pearson correlation coefficient was employed to assess criterion-related validity by determining the degree to which each item correlated with two generic items, namely, overall QOL and general health, which were used as the criteria. Furthermore, the corrected item-total correlations of the four subscales represent the relationships between each of the WHOQOL-BREF-THAI items and its own subscale, with that item deleted.

Convergent validity was assessed using the AVE. The AVE must be equal to or more than 0.50 to establish convergent validity, indicating that the variance of the construct accounts for more than 50% of its variation. ^37^ Discriminant validity was also used to determine whether the four WHOQOL-BREF domains were unique factors. Pearson’s correlation (r) values below 0.85 demonstrated discriminant validity between the variables. ^37^ The intercorrelations matrix and the temporal stability of the WHOQOL-BREF subscale scores were both investigated using Pearson correlations.

## Results

This study recruited 3,570 nursing students. The majority of those who completed the questionnaire (92.3%) were female, with a mean age of 20.29 (±2.12) years. More than two-thirds (72.6%) of these individuals lived on campus. About 28.4% of these participants reported alcohol consumption. Table 1 shows the characteristics of the nursing students (n = 3,570).

**Table 1.** Demographic characteristics of the participants (n = 3,570).

Characteristic		Mean ±SD or n (%)	
Gender	Male	375	7.7
	Female	3,295	92.3
Age in years	Mean (±SD)	20.29 (±2.12)	
	Median (min: max)	20 (18:35)	
Academic year	First year	1,208	33.8
	Second year	1,060	29.7
	Third year	761	21.3
	Fourth year	541	15.2
GPA	Mean (±SD)	3.18 (±0.37)	
	Median (min: max)	3.21(1.88:4.00)	
Residence	Live on campus	2,567	72.6
	Live off campus	969	27.4
Income	Mean (±SD)	5,191.42 (±2423.48)	
	Median (min: max)	5,000 (1,000:18,000)	
Smoking habit	No	3,442	96.4
	Yes	128	3.6
Alcohol consumption	No	2,557	71.6
	Yes	1,013	28.4

Note: SD = standard deviation.

Table 2 shows the results of the descriptive analysis for the 24 subscales of the WHOQOL-BREF. Each item had a range of 1-5 points, and no floor or ceiling effects were observed for any of the items. In addition, all items had polyserial item-rest correlation coefficients larger than 0.20, indicating their discriminative ability. In addition, Cronbach’s alpha values varied between 0.59 and 0.81 when each item was removed.

**Table 2.** Distribution of responses and discrimination of the items.

Item	Mean (±SD)	Response (%)					Item-rest correlation	Cronbach’s alpha if an item was deleted
		1	2	3	4	5		
1	3.39 (±0.91)	7.7	15.9	45.6	23.5	7.3	0.41	0.75
2	3.51 (±0.89)	13.4	36.0	40.0	9.2	1.4	0.42	0.79
3	2.72 (±0.93)	11.1	30.6	37.7	15.8	4.8	0.33	0.69
4	3.52 (±0.95)	8.2	24.2	37.6	21.1	8.9	0.50	0.79
5	3.09 (±0.97)	5.6	24.8	43.5	21.6	8.5	0.52	0.81
6	3.42 (±0.92)	1.4	14.2	40.8	30.3	13.3	0.67	0.78
7	3.26 (±0.81)	1.1	14.1	50.0	28.0	6.8	0.64	0.68
8	3.52 (±0.92	1.6	12.0	38.7	32.1	15.5	0.70	0.79
9	2.98 (±0.93)	1.7	11.3	37.8	31.6	17.8	0.66	0.81
10	3.10 (±0.93)	12.3	29.8	36.4	15.9	5.6	0.30	0.69
11	3.50 (±0.91)	8.7	23.4	37.6	20.4	9.9	0.50	0.81
12	3.59 (±0.93)	7.9	20.1	4.4	19.8	7.8	0.53	0.78
13	3.54 (±0.92)	1.4	7.0	27.9	30.6	33.2	0.72	0.68
14	3.78 (±0.89)	1.4	7.0	27.9	30.6	33.2	0.72	0.75
15	3.49 (±0.89)	1.4	11.9	40.7	31.6	14.4	0.74	0.82
16	3.53 (±0.91)	1.7	12.0	37.5	30.8	18.0	0.72	0.79
17	3.23 (±0.89)	2.4	14.9	47.1	25.2	10.4	0.63	0.65
18	3.47 (±0.91)	1.3	12.0	42.2	30.8	13.7	0.73	0.61
19	3.41 (±0.89)	1.1	12.1	42.8	31.6	12.4	0.72	0.59
20	3.08 (±0.89)	2.9	17.4	47.3	23.8	8.6	0.60	0.69
21	3.32 (±0.92)	1.7	13.1	43.8	29.4	12.0	0.73	0.80
22	3.24 (±0.91)	2.0	14.1	44.5	28.5	10.9	0.69	0.59
23	3.83 (±0.94)	7.9	22.8	35.0	20.2	14.1	0.66	0.66
24	3.75 (±0.91)	7.8	22.8	35.1	20.2	14.1	0.50	0.70
25	3.90 (±0.89)	1.3	7.0	27.9	30.6	33.2	0.72	0.58
26	3.60 (±0.89)	1.1	8.7	39.5	31.9	18.8	0.72	0.81

Following the random selection of 2,000 participants for EFA, scree plot and parallel analysis of the matrix revealed that a four-factor solution could be extracted. The matrix of rotational factor loading was statistically significant. The group 1 subsample yielded four components with eigenvalues greater than 1 in the initial analyses. The scree plot and the eigenvalue > 1 criterion revealed that the four factors resembled the original structure (eigenvalues: 10.52, 4.78, 1.52, and 1.34), and this model explained 75.6% of the total variance. The KMO measure verified the sampling adequacy (KMO = 0.97) and the significant result of Bartlett’s test of sphericity (p-value < 0.001), which indicated that the data in this study were suitable for factor analysis. Moreover, most of communalities were close to 1 (0.61-0.83), indicating that the model explains most of the variation in those variables. Table 3 shows the factor loadings for each WHOQOL-BREF item, with factor loadings greater than 0.50 indicating acceptable loading.

**Table 3.** Factor loading values for each item of the scale (n = 2,000).

Item	Factor loading ^a^				Communality
	Physical health	Psychological health	Social relationships	Environment	
Pain and discomfort	0.89				0.81
Energy and fatigue	0.88				0.81
Sleep and rest	0.59				0.83
Activities of daily living	0.79				0.75
Dependence on medication	0.55				0.78
Working capacity	0.55				0.79
Mobility	0.59				0.77
Positive feelings		0.79			0.86
Concentration		0.85			0.78
Self-esteem		0.86			0.83
Bodily appearance		0.87			0.73
Negative feelings		0.83			0.61
Spirituality		0.75			0.69
Personal relationships			0.72		0.91
Social support			0.71		0.90
Sexual activity			0.71		0.91
Physical safety & security				0.82	0.73
Home environment				0.80	0.70
Financial resources				0.83	0.68
Access to health services				0.84	0.76
Information				0.84	0.72
Leisure time				0.82	0.81
Physical environment				0.85	0.86
Transport				0.84	0.71
KMO	0.97				
Cronbach’s alpha	0.73	0.82	0.72	0.84	
Total of Cronbach’s alpha	0.94				
Cumulative %	75.6				

^a^ Extraction method: Principal component analysis.

**Figure 1.**
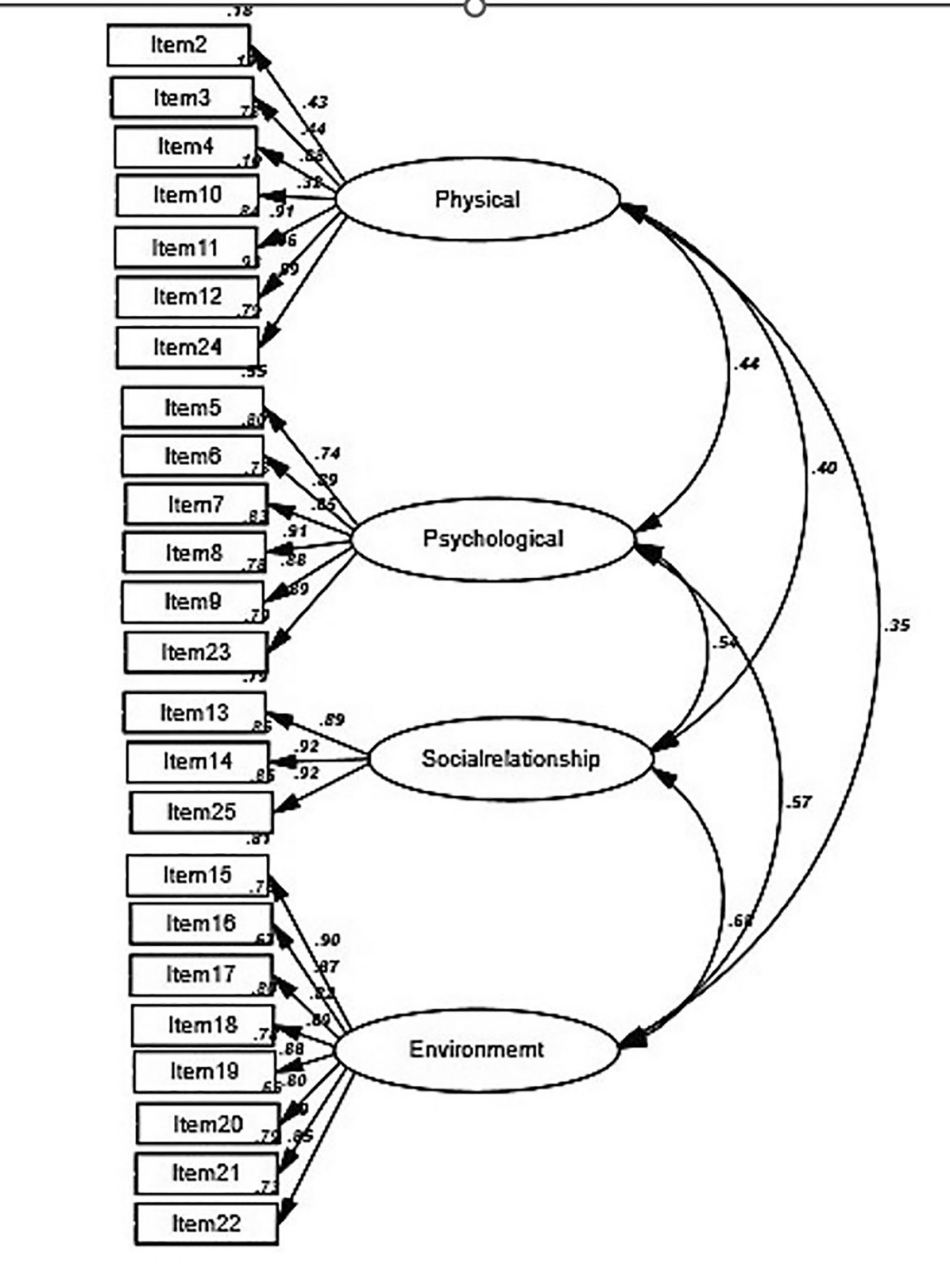
Four-domain model of the WHOQOL-BREF-THAI and standardized regression coefficients.

The WHOQOL-BREF measurement model, comprising 24 items distributed across four domains—physical health (7 items), psychological health (6 items), social relationships (3 items), and environment—was fitted using an unweighted least squares CFA. The correlation coefficients between the domains varied from 0.35 to 0.68, and the standardized regression weights ranged from 0.32 to 0.92 ( Figure 1). Based on the four specified fit criteria, the model demonstrated an acceptable fit to the data (χ ^2^ = 348.26/df = 113; RMSEA = 0.05; GFI = 0.97; and TLI = 0.91). Although the large sample size made the chi-square test inadequate for assessing model fit, other indices revealed that the model still fitted the data well. In addition, except for the factor-constrained items, all model items were significantly loaded onto their respective factors (all p-values < 0.05), with no significant tests remaining.

In all cases, the Pearson correlation coefficients between the WHOQOL-BREF domains ranged from 0.35 to 0.68, indicating fair to moderate correlations and indicating that these scales were moderately to highly discriminative.

The convergent validity of the modified WHOQOL-BREF measurement model was assessed in the context of this study using CR and AVE. In the current study, all subscales of the WHOQOL-BREF exhibited CR values exceeding the threshold of 0.70 (ranging between 0.76 and 0.95), indicating high internal consistency ( Table 4). This suggests that the items within each subscale cohesively measure the same underlying construct. AVE calculations showed that all WHOQOL-BREF subscales achieved convergence, with the following AVE values: physical health = 0.50, psychological health = 0.68, social relationships = 0.51, and environment = 0.69.

**Table 4.** Convergent validity and internal consistency of the WHOQOL-BREF.

WHOQOL-BREF domain	Composite reliability (CR)	Average variance extracted (AVE)	Cronbach’s alpha	Item-total correlation
Physical health	0.73	0.50	0.73	0.33-0.53
Psychological health	0.82	0.68	0.82	0.52-0.70
Social relationships	0.72	0.51	0.72	0.69-0.71
Environment	0.84	0.69	0.84	0.60-0.70
Total	-	-	0.94	0.33-0.71

According to the Cronbach’s alpha values, which were 0.94 for the overall scale, 0.73 for physical health, 0.82 for psychological health, 0.72 for social relationships, and 0.84 for environment, the WHOQOL-BREF exhibited adequate internal reliability as all values were above 0.70 ( Table 4). In addition, the CR values of the four domains, ranging from 0.76 to 0.95, indicated evidence of acceptable reliability. With a Cronbach’s alpha value of 0.84, the environment dimension demonstrated even greater consistency. These findings suggest that the items within these dimensions cohesively measure their intended constructs. Furthermore, the internal consistency of this scale was good, as evidenced by the item-rest correlations, which were higher than 0.30 for all four subscales, and the corrected item-rest correlations, which varied from 0.33 to 0.72 for the entire scale.

The strong magnitude of the correlations between all four domains indicated good discriminant validity ( Figure 1). Furthermore, r values between the variables were all smaller than 0.85, confirming their discriminant validity.

The inter-correlations matrix among the four subscales of the WHOQOL-BREF and overall QOL demonstrated strong inter-correlation values ( Figure 1). Notably, the subscales of environment and psychological health showed the strongest inter-correlation among the four domains (r = 0.68).

The correlation between each subscale item and the general categories, which are overall QOL (Q1) and general health (Q2), is significant (p-value < 0.001). Each individual question had a moderate-to-fair level of correlation with Q1 (0.22 ≤ r ≤ 0.52, p-value < 0.001) and Q2 (0.22 ≤ r ≤ 0.66, p-value < 0.001). According to the findings, each subscale item demonstrated a suitable degree of criterion-related validity, showing consistency in terms of direction and connection with the two criteria, as indicated in Table 5.

**Table 5.** Criterion-related validity of WHOQOL-BREF domains and items (n = 2,000).

Domain	Criterion-related validity	
	Overall QOL (Q _1_ ^a^)	General health (Q ^a^_2_)
Domain 1: Physical health		
Pain and discomfort	0.26 *	0.36 *
Energy and fatigue	0.44 *	0.52 *
Sleep and rest	0.24 *	0.22 *
Activities of daily living	0.22 *	0.45 *
Dependence on medication	0.23 *	0.26 *
Working capacity	0.25 *	0.27 *
Mobility	0.30 *	0.31 *
Domain 2: Psychological health		
Positive feelings	0.41 *	0.27 *
Concentration	0.52 *	0.39 *
Self-esteem	0.40 *	0.48 *
Bodily appearance	0.42 *	0.43 *
Negative feelings	0.41 *	0.47 *
Spirituality	0.33 *	0.66 *
Domain 3: Social relationships		
Personal relationships	0.30 *	0.32 *
Social support	0.29 *	0.59 *
Sexual activity	0.28 *	0.33 *
Domain 4: Environment		
Physical safety and security	0.40 *	0.66 *
Home environment	0.37 *	0.65 *
Financial resources	0.35 *	0.62 *
Access to health services	0.37 *	0.61 *
Information	0.35 *	0.59 *
Leisure time	0.37 *	0.61 *
Physical environment	0.38 *	0.58 *
Transport	0.27 *	0.53 *
All 24 WHOQOL-BREF items	0.46 *	0.27 *

^a^ Criterion-related validity refers to the strength of the Pearson correlation between each domain/its items and the two criterion items, i.e., overall QOL (Q1) and general health (Q2).

* p *-*value < 0.001.

## Discussion

To our knowledge, this is the first study that aims to examine the psychometric properties of the WHOQOL-BREF among nursing students. Overall, the current findings support the use of WHOQOL-BREF- THAI with Thai nursing students in northeast Thailand. The reliability and criterion-related validity were excellent, and the fit of the hypothesized four domains of QOL was acceptable. Furthermore, the item-scale correlation matrix for the WHOQOL-BREF measurement revealed that all items in the physical health (7 items), psychological health (6 items), social relationships (3 items), and environment (8 items) domains had extremely high correlation coefficients with their respective domains. This result is comparable to a prior study in Asia that aimed to evaluate the psychometric properties of the WHOQOL-BREF questionnaire among disabled students in Malaysian higher learning institutions. ^15^ Moreover, the CFA results from a study of adults with disabilities and adults from the general population in Singapore also supported the four domains. ^18^ In addition, most of the communality values were close to 1, indicating that the extracted factors explained most of the variance in each individual item. ^38^ These results were in line with a study on the validity and reliability of the WHOQOL-BREF in a pregnant population. ^39^

Concerning data distribution, no floor or ceiling effects were observed in any of the items. These findings suggest that the WHOQOL-BREF instrument can effectively measure the responsiveness to change in nursing students with elevated scale scores. ^40^ Item analysis revealed strong discrimination indices for each scale. All item-total correlations were highly and positively correlated with the total score, demonstrating the scale’s homogeneity. These indices revealed that by utilizing the Thai version of the WHOQOL-BREF items, high and low scores on this scale could be distinguished. These results were comparable with a previous study that aimed to evaluate the psychometric properties of this tool in the Vietnamese young adult population. ^28^

According to the CFA results, the goodness-of-fit indices were acceptable, and the four main factor structures with eigenvalues greater than 1 explained 75.6% of the total variance. The WHOQOL-BREF was confirmed in this sample to be consistent with earlier WHOQOL-BREF research. ^13, 17, 23, 24, 26^ Although previous research suggested that WHOQOL-BREF factor extraction could yield different solutions in some instances, ^25, 27, 29^ the four-factor solution was considered essential for a better model fit, as the former has disadvantages in terms of inter-item correlations. Similar research in China supported the use of the WHOQOL-BREF for medical students. ^41^ Another finding from our study that demonstrates the structural validity of the tool is that the correlation values between its sub-dimensions were positive and significant across all dimensions. These results are comparable with a previous study that aimed to evaluate the psychometric properties of this tool in an adult population. ^18^

The Cronbach’s alpha coefficients for the total score and the four domain scores ranged from 0.85-0.97, and the CR values above 0.7 revealed that the WHOQOL-BREF-THAI scores and subscales had good internal consistency, comparable to the Cronbach’s alpha values of the original scale. ^1^ This coefficient ranged from good to excellent in previous studies involving both nonclinical and clinical adult samples. ^13, 15– 17, 19, 23, 42^ Hence, the data collectively show that the WHOQOL-BREF-THAI demonstrates strong internal consistency across a variety of demographics and languages. Considering the internal consistency scores of the WHOQOL-BREF domains, the environmental domain had the highest Cronbach’s alpha, which was consistent with a previous study that examined the validity and reliability of the WHOQOL-BREF in Vietnamese young adults. ^28^ The low Cronbach’s alpha in the social relationships domain should be interpreted with caution due to the use of only three item scores, instead of the required four, for internal reliability assessment. ^35^ Moreover, the test-retest reliability results of this study, which classify the WHOQOL-BREF-THAI as an excellent tool, are consistent with a previous study. ^20^

The association between the mean scores of each item/domain scores with the single item of overall QOL was used to evaluate the criterion-related validity. As hypothesized, all domains showed significant positive correlations with items on overall QOL and general health. The results show that all items in the scale and its four domains have adequate criterion-related validity, with significant relationships with the two criteria. This result is in line with the study that aimed to examine the performance of the WHOQOL-BREF-THAI in assessing the QOL among Thai college students. ^13^ Furthermore, our findings showed that the psychological health domain had the greatest impact on overall QOL, followed by the environment domain. Similarly, the environment domain had the greatest impact on health satisfaction, followed by the psychological health domain. This outcome is consistent with earlier research findings, ^18^ indicating the importance of psychological well-being for overall QOL and health. The validity findings show that the WHOQOL-BREF-THAI is well validated for Thai nursing students. These findings are in line with previous studies, ^13, 39^ which also found that this tool was well validated for both Thai college students ^13^ and the pregnant population. ^39^

To assess convergent validity, the AVE calculation of all four subscales was used to evaluate the WHOQOL-BREF. The results demonstrated that the AVE for all subscales was greater than 0.50, which corresponded to the convergent validity acceptance criterion. In addition, convergent validity was demonstrated by the significant positive correlations between the scale domains and overall QOL, as well as the positive correlations among all four WHOQOL-BREF domains. Moreover, several international studies have found the WHOQOL-BREF to have convergent and discriminant validity. ^1, 13, 24, 26^ These findings confirm the validity and reliability of the WHOQOL-BREF Thai version as an instrument for measuring positive emotional states. This implies that this scale could be beneficial for assessing QOL in healthcare personnel and health science students, such as nursing students.

The results of the intercorrelations matrix between the four WHOQOL-BREF subscales and overall QOL suggested that there were strong item-domain correlations, particularly between the physical health and environment domains (r = 0.68). This may indicate that the good environment provided to nursing students is associated with improved physical health. This finding is comparable with prior study that aimed to evaluate psychometric properties of the WHOQOL-BREF questionnaire among disabled students. ^15^

### Strengths and limitations

This study is unique in that it examines the WHOQOL-BREF-THAI in a large sample of Thai undergraduate nursing students. Because the participant-to-item ratio was adequate, the criteria for component analysis were met, and bias resulting from the number of observations was reduced.

Our research has some limitations because our sample was made up entirely of nursing students. Because of the diversity of characteristics, this sample cannot be accurately generalized to other non-clinical undergraduate students, as they may differ in terms of socioeconomic status, freedom, and health. Since the research was cross-sectional, the data could not demonstrate test-retest reliability over time.

## Conclusion

According to our findings, the WHOQOL-BREF is a reliable and valid tool for healthcare providers to use for assessing QOL among Thai nursing students. QOL is likely to have an impact on their future clinical success. Therefore, the WHOQOL-BREF can be an essential tool for researching the factors influencing the QOL of nursing students, with implications for nursing education.

### Ethical considerations

This research was approved by the Roi-Et Rajabhat University Ethics Committee for Human Research (Reference No. RERU-EC 013/2567). The date of approval was the 15th of January 2024. Ethics Committees for Human Research, based on the Declaration of Helsinki and the ICH Good Clinical Practice Guidelines Written informed consent was obtained from all patients. Participants were informed that they could withdraw from the study at any time without providing a reason. The confidentiality of participant data was ensured throughout the entire study.

## Data Availability

Figshare: Validation of the Thai version of the World Health Organization’s Quality of Life Scale (WHOQOL-BREF-THAI) among Thai Nursing Students in Northeast Thailand: A multi-canter study. The data provide do not have subheading and this project contains the following underlying data Figshare:, DOI: https://doi.org/10.6084/m9.figshare.28136420.v2 ^43^ The project contains the following underlying data:
• Data.xlsx Data are available under the terms of the Creative Commons Attribution 4.0 International License (CC-BY 4.0). Figshare: Validation of the Thai version of the World Health Organization’s Quality of Life Scale (WHOQOL-BREF-THAI) among Thai Nursing Students in Northeast Thailand: A multi-canter study. The data provide do not have subheading and this project contains the following underlying data Figshare:, DOI: https://doi.org/10.6084/m9.figshare.28136420.v2 ^43^ The project contains the following extended data:
• Ethical consideration • Questionnaires (including the English version of WHOQOL-BREF) Data are available under the terms of the Creative Commons Attribution 4.0 International License (CC-BY 4.0).
