# Supplementary material for: Validation of the Thai version of the World Health Organization’s Quality of Life Scale (WHOQOL-BREF-THAI) among Thai nursing students in northeast Thailand: A multi-centre study
Source: F1000Res. 2025 Jul 22;14:241. Originally published 2025 Feb 27. [Version 3] doi: 10.12688/f1000research.161251.3 (PMC12770885; doi:10.12688/f1000research.161251.3)
Supplement: Peer review history

# Reviewer report: Reviewer response for version 3

**DOI:** 10.5256/f1000research.185381.r442205

**Published:** 2026-01-05

**Authors:** Evija Nagle

**Affiliations:** 1 Rīga Stradiņš University, Rīga, Latvia

The submitted manuscript presents a large-scale, multi-center psychometric validation study of the WHOQOL-BREF-THAI in a population of nursing students in northeastern Thailand. The research topic is relevant and topical, and the sample size is excellently suited for factor analysis, allowing for the reasonable use of split-sample exploratory (EFA) and confirmatory factor analysis (CFA). Overall, the study provides a significant empirical contribution to the use of quality-of-life assessment instruments in the context of nursing education.

 From a methodological perspective, random allocation of the sample for EFA (n = 2000) and CFA (n = 1570) is appropriate and consistent with best practice in psychometric research. The reported KMO scores, Bartlett's test of sphericity, factor loadings, and total variance explained indicate very good data suitability for factor analysis. Internal consistency measures (Cronbach α and composite reliability) as well as convergent validity estimates (AVE) for all subscales are at an acceptable or high level, which generally confirms the reliability of the instrument.

 At the same time, there are several aspects that should be clarified for the manuscript to fully comply with high-level psychometric reporting standards. First, although the term “exploratory factor analysis” is used in the text, principal component analysis (PCA) was used to obtain the factors. This methodological choice should be clearly acknowledged and theoretically justified, considering the differences between PCA and total factor analysis in the context of construct validation. Second, the choice of the WLSMV estimation method used in the CFA analysis should be more clearly justified in relation to the nature of Likert-type ordinal data to improve the transparency and reproducibility of the study.

 The description of the cultural adaptation process in the manuscript is limited. Given that the WHOQOL-BREF-THAI is an adapted instrument, it would be useful to briefly but clearly describe the previous translation, cultural adaptation and content validation steps, even if they were carried out in other studies. This would strengthen the argument for construct equivalence in the nursing student population. Furthermore, the discriminant validity assessment is mainly based on interfactor correlations; the inclusion of additional criteria, such as the Fornell–Larcker approach, would further strengthen the validity evidence.

 In the discussion section, the results are generally well integrated into the international literature, however, it is recommended to expand the limitations section by more clearly emphasizing the cross-sectional design, the use of self-report data and the lack of test–retest reliability. Even if longitudinal data are not available in this study, these aspects should be clearly highlighted as directions for future research. It would also be useful to discuss more broadly the practical applications of the instrument – ​​for example, in screening student well-being, institutional monitoring or in planning targeted support intervention programs in nursing education.

 In conclusion, the study is scientifically sound and based on a strong empirical foundation. By introducing methodological refinements and deepening the analysis of cultural adaptation and constraints, the manuscript would be significantly strengthened both methodologically and practically.

Is the work clearly and accurately presented and does it cite the current literature?

Partly

If applicable, is the statistical analysis and its interpretation appropriate?

Partly

Are all the source data underlying the results available to ensure full reproducibility?

Yes

Is the study design appropriate and is the work technically sound?

Yes

Are the conclusions drawn adequately supported by the results?

Yes

Are sufficient details of methods and analysis provided to allow replication by others?

Partly

Reviewer Expertise:

NA

I confirm that I have read this submission and believe that I have an appropriate level of expertise to confirm that it is of an acceptable scientific standard, however I have significant reservations, as outlined above.

# Reviewer report: Reviewer response for version 2

**DOI:** 10.5256/f1000research.182565.r395384

**Published:** 2025-07-07

**Authors:** Edison Luiz Devos Barlem

**Affiliations:** 1 Universidade Federal do Rio Grande, Rio Grande, Brazil

Thank you for submitting the manuscript entitled *"Validation of the Thai version of the World Health Organization’s Quality of Life Scale (WHOQOL-BREF-THAI) among Thai nursing students in northeast Thailand: A multi-centre study."* After a thorough review, we acknowledge that this study presents a relevant and timely contribution by seeking to validate a culturally adapted version of the WHOQOL-BREF among nursing students—a population often exposed to stressors that directly impact their quality of life and academic performance. The methodological design is robust, and the sample size is adequate, with proper use of statistical techniques for both exploratory and confirmatory factor analyses. Nevertheless, we identified areas that require improvement, which are detailed below.

 To begin with, the title is well formulated and accurately reflects the study's scope. The abstract follows the conventional structure, though it remains overly synthetic in some essential areas. We recommend expanding the methods section in the abstract and including core statistical indicators, such as model fit values and key numerical findings. It is also important to mention the psychometric tools applied (EFA, CFA, AVE, CR) to ensure clearer communication of the results.

 The objective is pertinent and aligns with the justification provided, but we suggest reformulating it to ensure greater clarity and methodological specificity. For instance: *“To assess the psychometric properties (construct validity, internal consistency, and convergent/discriminant validity) of the Thai version of the WHOQOL-BREF among nursing students in northeastern Thailand.”* This revised version more accurately reflects the methodological nature of the study.

 The introduction provides a solid overview of the quality of life (QoL) concept, based on recent and relevant literature. However, we encourage a deeper conceptual discussion, especially drawing on classical WHO documents and leading authors on QoL assessments in educational and cultural contexts. Additionally, although the manuscript refers to cultural variability in the WHOQOL-BREF’s factor structure, this discussion would benefit from a more critical perspective, addressing the theoretical and methodological implications of cross-cultural validity.

 The methods section is clearly articulated and well developed, detailing the sampling procedures, inclusion and exclusion criteria, and appropriate statistical approaches. The decision to split the sample for EFA and CFA is methodologically sound. However, it is important to specify the statistical software used, justify the choice of WLSMV estimation in relation to the ordinal nature of the data, and discuss the criteria used to exclude cross-loading items. We also suggest explaining whether normality tests were performed and reflecting on potential bias from using self-administered questionnaires.

 The results are clearly presented, with relevant tables and statistical indicators. The factor analysis is consistent with the objectives, and the fit indices support the validity of the four-domain model. Nevertheless, we recommend that the authors explore potential associations between sociodemographic variables and QoL domains using additional analyses (e.g., ANOVA or multiple regression). Such analysis would provide further insight into determinants of QoL among nursing students.

 The discussion is well structured and draws appropriate comparisons with previous studies. However, it lacks critical reflection on the study’s own limitations—such as the cross-sectional design, potential social desirability bias, and the lack of test-retest reliability assessment. It would be valuable to expand on the practical implications of the validated instrument for nursing education management, institutional screening, and mental health support programs. We suggest including this in a dedicated subsection.

 The conclusions are consistent with the findings but could be strengthened by proposing the use of the instrument in intervention contexts, longitudinal evaluations, and institutional policies for student support. Emphasizing the practical contributions of the study would enhance its scientific and social impact.

 The references are mostly recent and relevant. However, we recommend including additional sources such as systematic reviews and international guidelines on cross-cultural validation of psychometric instruments.

 In summary, this is a relevant study with the potential to contribute meaningfully to research on mental health and nursing education. Nonetheless, to fully meet the standards of high-impact international journals, we recommend a **major revision**, incorporating the suggestions detailed above. We remain available to re-evaluate the manuscript should the authors decide to submit a revised version.

Is the work clearly and accurately presented and does it cite the current literature?

Partly

If applicable, is the statistical analysis and its interpretation appropriate?

Partly

Are all the source data underlying the results available to ensure full reproducibility?

Yes

Is the study design appropriate and is the work technically sound?

Yes

Are the conclusions drawn adequately supported by the results?

Yes

Are sufficient details of methods and analysis provided to allow replication by others?

Partly

Reviewer Expertise:

Nursing

I confirm that I have read this submission and believe that I have an appropriate level of expertise to confirm that it is of an acceptable scientific standard, however I have significant reservations, as outlined above.

# Response

**Published:** 2025-07-19

**Authors:** Ueamporn Summart

**Affiliations:** Faculty of Public Health, Khon Kaen University, Khon Kaen, Thailand

Thank you for all of your suggestions and comments for improving our manuscripts. I attempted to edit and revise our manuscripts followed your suggestions; however, our study have some limitation about the data collection, so we cannot  explore potential associations between sociodemographic variables and QoL domains using additional analyses (e.g., ANOVA or multiple

 regression). Such analysis would provide further insight into determinants of QoL among nursing

 students.

 1) abstracts: Thank you for your suggestions. We edited our abstract following your comments, including objective, methods, and results.

 2) We specify the statistical software used and the normality test for supporting our statistical analysis.

 3) We also added the systematic reviews of WHOQOL questionnaires and discussed with the community for supporting our psychometric properties of this measurement.

 4) We added our limitations due to the cross-sectional design and lack of test-retest into the limitation sections.

 5) We revised our conclusions with suggestions for longitudinal studies for better outcomes.

 Finally, we appreciated your suggestions for improving our manuscripts. Please consider our revised manuscript.

 Best regards,

 Ueamporn Summart

# Reviewer report: Reviewer response for version 2

**DOI:** 10.5256/f1000research.182565.r386867

**Published:** 2025-06-14

**Authors:** Maria Lúcia do Carmo Cruz Robazzi

**Affiliations:** 1 University of São Paulo, São Paulo, Brazil

I have already reviewed the manuscript and the recent changes, and I **consider the article approved**. The revisions made are appropriate, and I see no need for further modifications or to change my previous review status.

Is the work clearly and accurately presented and does it cite the current literature?

Yes

If applicable, is the statistical analysis and its interpretation appropriate?

Yes

Are all the source data underlying the results available to ensure full reproducibility?

Yes

Is the study design appropriate and is the work technically sound?

Yes

Are the conclusions drawn adequately supported by the results?

Yes

Are sufficient details of methods and analysis provided to allow replication by others?

Yes

Reviewer Expertise:

Nursing; Ocuupational Health

I confirm that I have read this submission and believe that I have an appropriate level of expertise to confirm that it is of an acceptable scientific standard.

# Reviewer report: Reviewer response for version 1

**DOI:** 10.5256/f1000research.177256.r375812

**Published:** 2025-05-21

**Authors:** Maria Lúcia do Carmo Cruz Robazzi, Fabio de Souza Terra

**Affiliations:** 1 University of São Paulo, São Paulo, Brazil; 2 School of Nursing, Federal University of Alfenas, Alfenas, Minas Gerais, Brazil

This study, which validated the Thai version of the WHOQOL-BREF in a robust sample of 3,570 nursing students from northeastern Thailand, presents relevant results regarding the psychometric properties of the instrument in this specific population. The analysis identified a four-domain structure with 24 items, demonstrating good internal consistency (alpha > 0.70) and convergent validity (AVE > 0.50). The authors pertinently conclude that the WHOQOL-BREF-THAI is a valid and reliable tool for assessing the quality of life of these students, with potential application in nursing research and education.

 Considering the validation context – nursing students in all four years of undergraduate study in northeastern Thailand – the study proves to be interesting and necessary. The cited literature is relevant to the topic. However, it is observed that only 32.55% of the 43 references presented are from the last five years. It is therefore suggested that the authors seek to include a greater number of updated references, aiming to reach at least 50% of more recent citations.

 Although subsequent and suggested studies in this review do not focus on the validation of the WHOQOL-BREF in nursing students or analyze other instruments, some may enrich the discussion of the present work. The following references, for example, could be considered in the Introduction, Method, or Discussion to increase the percentage of recent studies:

 ( refer 1, 2 , 3 and 4 )

 Summary: The objective presented in the summary is consistent with that of the body of the text. The summary is clear, and the conclusion adequately responds to the proposed objective.

 Introduction: Well-presented and clear, the introduction directs the reader to the gap in the literature regarding the psychometric properties of the WHOQOL-BREF in Thai nursing students, clearly establishing the main objective of the study. It is important to note that the authors used the WHO's concept of quality of life (QOL), the organizing principle of the original WHOQOL, which in its construction used this same concept of QOL as a theoretical framework.

 Method: The study design is detailed enough to allow for replication by other researchers. However, as it is a validation study, it is suggested that it also be characterized as a “methodological study.” The statistical analysis employed is appropriate, and the sample of 3,570 Thai nursing students was adequately divided for the exploratory (n=2000) and confirmatory (n=1570) analyses.

 Results: The results are presented clearly through five tables and one figure, highlighting the main findings that address the study's objective, which is expected for research of this nature.

 Discussion: The discussion is adequate and based on relevant literature, although the inclusion of more recent studies could enrich it.

 Strengths and Limitations: Adequately presented. However, if the authors aimed to study only nursing students, we do not understand this element as a limitation; other authors and new studies can carry out the validation process with different students.

 Conclusions: The conclusions are consistent with the objective and based on the results.

 Data Availability: The data is accessible in the Figshare repository.

 In summary, this study significantly contributes to the understanding of the psychometric properties of the WHOQOL-BREF in Thai nursing students. The suggested updating of references may further strengthen the theoretical foundation and the discussion of the results.

Is the work clearly and accurately presented and does it cite the current literature?

Yes

If applicable, is the statistical analysis and its interpretation appropriate?

Yes

Are all the source data underlying the results available to ensure full reproducibility?

Yes

Is the study design appropriate and is the work technically sound?

Yes

Are the conclusions drawn adequately supported by the results?

Yes

Are sufficient details of methods and analysis provided to allow replication by others?

Yes

Reviewer Expertise:

Nursing; Ocuupational Health

We confirm that we have read this submission and believe that we have an appropriate level of expertise to confirm that it is of an acceptable scientific standard, however we have significant reservations, as outlined above.

## References

[1] : The Lifestyle and Quality of Life Among Nursing Students. *J Holist Nurs* .2025;43(1) : 10.1177/08980101241292208 18-25 10.1177/08980101241292208 39474641

[2] : Assessing the quality of life among African medical and health science students using the WHOQOL-BREF tool. *PeerJ* .2025;13: 10.7717/peerj.18809 e18809 10.7717/peerj.18809 39931073 PMC11809301

[3] : The Psychometric Properties of the World Health Organization Quality of Life-BREF Questionnaire in College Students. *J Nurs Meas* .2024;32(2) : 10.1891/JNM-2022-0062 256-266 10.1891/JNM-2022-0062 37348885

[4] : Psychometric properties of the Burnout Assessment Tool - General version in nursing workers. *Rev Lat Am Enfermagem* .2025;33: 10.1590/1518-8345.7367.4425 e4425 10.1590/1518-8345.7367.4425 39907387 PMC11789741

# Response

**Published:** 2025-05-21

**Authors:** Ueamporn Summart

**Affiliations:** Faculty of Public Health, Khon Kaen University, Khon Kaen, Thailand

Thank you for all of your suggestions. After we considered all of your suggestions, we agreed with your opinions  to edit and revise our manuscript as follows:

 1) We edited our citation following your suggestion in the introduction part and discussion part.

 2) We edited our study design into "a methodological study." Please see our revised manuscript.

 3) Limitation: Thanks for your suggestions. We deleted this statement from this part and added another limitation regarding data being collected at a single point in time.

# Response

**Published:** 2025-05-21

**Authors:** Ueamporn Summart

**Affiliations:** Faculty of Public Health, Khon Kaen University, Khon Kaen, Thailand

I would like to express my gratitude for your diligent efforts to comment on and suggestions for improving our manuscript. We appreciated all of this and attempted to edit this manuscript following your comments. If you have any comments, we do not hesitate to edit our manuscripts following your comments and send them back to your process as fast as possible.
